# Bilevel Optimization for ISAC Systems with Proactive Eavesdropping Capabilities

**DOI:** 10.3390/s25134238

**Published:** 2025-07-07

**Authors:** Tingyue Xue, Wenhao Lu, Mianyi Zhang, Yinghui He, Yunlong Cai, Guanding Yu

**Affiliations:** College of Information Science and Electronic Engineering, Zhejiang University, Hangzhou 310027, China; 3220103218@zju.edu.cn (T.X.); 3220103223@zju.edu.cn (W.L.); mianyi_zhang@zju.edu.cn (M.Z.); 2014hyh@zju.edu.cn (Y.H.); ylcai@zju.edu.cn (Y.C.)

**Keywords:** integrated sensing and communication, proactive eavesdropping, radar detection, bilevel optimization

## Abstract

Integrated sensing and communication (ISAC) has attracted extensive attention as a key technology to improve spectrum utilization and system performance for future wireless sensor networks. At the same time, active surveillance, as a legitimate means of surveillance, can improve the success rate of surveillance by sending interference signals to suspicious receivers, which is important for crime prevention and public safety. In this paper, we investigate the joint optimization of performance of both ISAC and active surveillance. Specifically, we formulate a bilevel optimization problem where the upper-level objective aims to maximize the probability of successful eavesdropping while the lower-level objective aims to optimize the localization performance of the radar on suspicious transmitters. By employing the Rayleigh quotient, introducing a decoupling strategy, and adding penalty terms, we propose an algorithm to solve the bilevel problem where the lower-level objective is convex. With the help of the proposed algorithm, we obtain the optimal solution of the analog transmit beamforming matrix and the digital beamforming vector. Performance analysis and discussion of key insights, such as the trade-off between eavesdropping success probability and radar localization accuracy, are also provided. Finally, comprehensive simulation results validate the effectiveness of our proposed algorithm in enhancing both the eavesdropping success probability and the accuracy of radar localization.

## 1. Introduction

The field of integrated sensing and communication (ISAC) marks a major advancement in wireless technology, combining wireless communication and radar sensing capabilities to enhance spectral efficiency and improve spatial–temporal resolution of signals. This integration is particularly crucial with the advent of future 6G technologies, where ISAC plays a pivotal role in high-speed data transmission and precise monitoring, especially in applications such as autonomous driving, wireless sensing and intelligent transportation systems [1]. However, in the realm of wireless communication, the open nature of the wireless medium makes it inherently susceptible to security vulnerabilities. Proactive eavesdropping, a legitimate and strategic form of surveillance, has become an essential capability for government agencies. Unlike passive eavesdropping, which can be limited by geographical factors or channel conditions, proactive eavesdropping involves the eavesdropper actively transmitting interference signals towards the target receiver to enhance the chances of successful interception. This approach holds practical application prospects, such as in vehicles or drones equipped with legal eavesdropping devices [2].

Building upon this, several studies have examined the integration of security measures within ISAC systems, addressing the simultaneous optimization of both communication and sensing functionalities. The authors of ref. [3] explore the use of Reconfigurable Intelligent Surfaces (RISs) to enhance the physical layer security (PLS) of wireless systems with ISAC. The paper discusses how RISs can mitigate threats like eavesdropping by intelligently manipulating the propagation of signals. However, while it emphasizes communication security, the interaction between communication security and radar sensing within ISAC systems still remains unexplored. Additionally, the authors of ref. [4] focus on a secure cell-free ISAC system, where both communication and sensing are subject to eavesdropping. The authors optimize beamforming strategies to ensure the security of both communication and sensing tasks. However, they treat these two objectives separately, making it difficult to efficiently coordinate the resources and balance both goals. In parallel, the authors of ref. [5] investigate a full-duplex ISAC system, where they jointly optimize beamforming and artificial noise to secure communications against eavesdroppers while simultaneously sensing them. However, their defensive framework aims to guarantee secrecy rates, which contrasts with our goal of proactively maximizing the eavesdropping probability. Additionally, the authors of ref. [6] study the integration of communication and sensing in ISAC networks for 6G. While it highlights the challenges of securing both communication and sensing functionalities, it does not provide a comprehensive solution for jointly optimizing both objectives. The authors suggest that while communication security and sensing can complement each other, an optimal framework to manage both simultaneously is still lacking.

Therefore, although these works address various aspects of ISAC security, they generally treat communication and sensing security as independent objectives. This approach may lead to difficulties in effectively balancing the dual goals, especially in mission-critical scenarios where both communication and sensing accuracy are essential. In particular, existing methods fail to efficiently allocate resources between eavesdropping prevention and radar localization, making it challenging to optimize both aspects simultaneously.

To address these limitations, bilevel optimization steps in as a powerful tool for solving multi-objective optimization problems [7]. In recent years, bilevel optimization has been adopted in several communication systems, particularly in the context of ISAC. For example, He et al. [8] utilize the bilevel optimization to optimize communication quality-of-service (QoS) and radar detection simultaneously, with the upper level focusing on communication performance and the lower level handling radar interference. Similarly, in [9], the authors consider an RIS-assisted SWIPT system, where bilevel optimization separates communication and power harvesting into two levels—maximizing information delivery at the upper level and harvested power at the lower level. This approach effectively balances the two objectives, optimizing overall system performance.

Building on these insights, our paper introduces a bilevel optimization approach that effectively coordinates communication security and radar sensing in ISAC systems. Specifically, we propose a system where a full-duplex legitimate monitor, equipped with a large-scale antenna array, uses hybrid beamforming to simultaneously perform proactive eavesdropping and radar sensing. The optimization focuses on maximizing the eavesdropping success probability while ensuring high radar localization accuracy. To achieve this, we first transform the eavesdropping success probability into a deterministic signal-to-interference-plus-noise ratio (SINR) difference and simplify the problem using Rayleigh quotient transformation. We then decouple the optimization problem and introduce penalty terms to handle non-convex components. The solution is derived by optimizing the beamforming vectors for both eavesdropping and radar tasks under certain system constraints. Furthermore, we discuss the simulation results, which demonstrate the effectiveness of our proposed approach in balancing both eavesdropping and radar performances.

We summarize our main contributions of this paper as follows:

• We present a comprehensive system model for proactive ISAC surveillance, characterizing the distinct signal flows for eavesdropping, radar sensing, and jamming within a unified full-duplex architecture.
• We formulate a mathematical optimization problem that maximizes the eavesdropping success probability while maintaining radar positioning accuracy. To solve this problem, we introduce a decoupling framework with penalty terms to handle non-convex components, enabling effective bilevel optimization of the problem’s convex objectives.
• We conduct a detailed analysis of the influence of factors such as noise power and antenna number on both the upper and lower-level objectives. Through simulations, we demonstrate that our bilevel algorithm not only achieves high radar localization accuracy but also maintains a comparable eavesdropping probability to existing benchmark methods.

The rest of this paper is structured as follows. Section 2 presents the system model and formulates the mathematical optimization for integrated surveillance and radar sensing. In Section 3, the optimal algorithm is introduced for dealing with the formulated problem. In Section 4, numerical results are presented to validate the effectiveness of the proposed algorithm. Finally, Section 5 concludes the whole paper.

Notations: In this paper, scalars, vectors, and matrices are denoted by lowercase letters (e.g., *x*), bold lowercase letters (e.g., x), and bold uppercase letters (e.g., X), respectively. The operators ${\left(\cdot \right)}^{*}$, ${\left(\cdot \right)}^{T}$, and ${\left(\cdot \right)}^{H}$ represent the complex conjugate, transpose, and Hermitian (complex conjugate transpose), respectively. Given a matrix A, the notation $∥A∥$ stands for its Frobenius norm. The set $C^{m\times n}$ denotes the space of complex matrices of size m×n, and I stands for the identity matrix. The notation CN(μ,Σ) indicates a circularly symmetric complex Gaussian distribution with mean vector μ and covariance matrix Σ. The expectation and probability operators are denoted by E{·} and P(·), respectively.

## 2. System Model and Problem Formulation

In this section, we present the system model for wireless legitimate surveillance with radar capabilities and establish the optimization problem.

### 2.1. System Model

**Figure 1:**
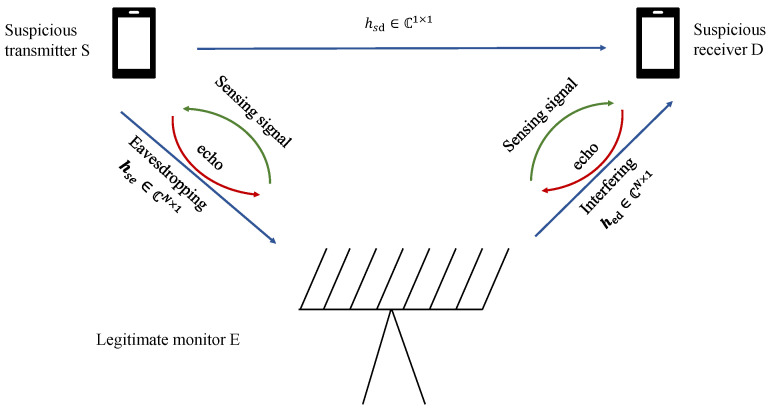
A legitimate surveillance system with radar function.

We consider a legitimate surveillance system that includes a suspicious transmitter *S*, a suspicious receiver *D*, and a legitimate monitor *E* as shown in Figure 1 [10]. It is important to note that the term “legitimate monitor” as used in this paper presupposes that the operating entity has been granted the legal authority to conduct such proactive jamming and surveillance activities. An analysis of the specific national statutes and regulatory frameworks that grant this authority—such as 47 U.S.C. § 333 of the Communications Act in the United States [11] or the Regulation of Investigatory Powers Act 2000 in the United Kingdom [12]—is beyond the technical scope of this work. Our focus remains on the signal processing and optimization challenges that arise under the fundamental assumption of a legally sanctioned operation. In this system, the transmitter S and suspicious receiver *D* are each equipped with a single antenna to transmit and receive the signals. The legitimate monitor *E* is equipped with *N* antennas and *M* radio frequency (RF) chains (M≪N), where *N* antennas are used for both transmitting and receiving signals. *E* intends to actively eavesdrop on suspicious transmissions between transmitter *S* and receiver *D*. To enhance the eavesdropping performance, *E* operates in the full-duplex (FD) mode, allowing it to simultaneously eavesdrop on the communication while interfering with the transmission between *S* and *D*. This interference is intentionally introduced by *E* to degrade the communication quality at *D*, which results in a lower SINR at *D* and, consequently, improves *E*’s ability to eavesdrop on the transmission. By reducing *D*’s SINR, *E* effectively boosts its own SINR and enhances eavesdropping performance.

Additionally, since the legitimate monitor can make contributions to legitimate monitoring in all kinds of ways, such as tracking suspicious users, detecting potential obstacles, or assisting in communication, we assume that the legitimate monitor operates as a phased-array radar, continuously transmitting detection signals in *N* directions of 180° during the scanning period. Each of these directions corresponds to one of the *N* beams generated by *E*’s radar system. Let $h_{se}\in C^{N\times 1},h_{sd}\in C^{1\times 1}$, and $h_{ed}\in C^{N\times 1}$ denote the eavesdropping channel, suspicious channel, and interference channel, respectively.

Regarding the transmitting module, we consider two types of signals: the suspicious signal and the interference signal. The suspicious signal is the communication signal transmitted from the suspicious transmitter *S* to the suspicious receiver *D*, which *E* is trying to eavesdrop on, and this signal can be written as ${\tilde{U}}_{n}^{H}h_{se}\sqrt{p_{s}}s\left(l\right)$, where s(l) is the baseband signal transmitted by *S*, $p_{s}$ is the transmit power, $h_{se}$ is the channel from *S* to *E*, and ${\tilde{U}}_{n}$ is the analog receive beamforming matrix at direction $\theta_{n}$. This is the target signal that *E* aims to recover during eavesdropping. The interference signal, on the other hand, is intentionally generated by *E* to degrade *D*’s reception quality. The legitimate monitor detects *N* directions consecutively in one scan cycle, probing one direction at a time. One RF chain is used for radar detection in each direction, while the remaining M−1 RF chains are used to generate interference to disturb *D*’s signal reception. Upon detecting the *n*-th direction, the transmitted signal at angle $\theta_{n}$ can be written as

(1) $$\begin{array}{c} x_{n}\left(l\right)=U_{n}p_{n,l}c_{n}\left(l\right), \end{array}$$

where $c_{n}\left(l\right)$ refers to the transmitted signal sequence with $E\{|c_{n}\left(l\right){|}^{2}\}=1$. This signal sequence serves as the common carrier for both the radar detection and interference signals. By sharing the same waveform, *E* is able to perform both functions simultaneously while efficiently utilizing its limited RF chains. The analog beamforming matrix $U_{n}=\left[u_{n,1},u_{n,2},\ldots ,u_{n,M}\right]\in C^{N\times M}$ determines the spatial direction of signal transmission, while the digital transmit beamforming vector $p_{n,l}={\left[p_{n,l,1},p_{n,l,2},\ldots ,p_{n,l,M}\right]}^{T}\in C^{M\times 1}$ adjusts the power and phase of each stream at a specific time index *l*. The signal index l=1,2,…,L denotes the time slot within a single beamforming direction during a full radar scan cycle. In other words, for every direction $\theta_{n}$, the monitor transmits *L* symbols before switching to the next. Together, the matrix $U_{n}$ and the potentially time-varying vector $p_{n,l}$ form the hybrid beamformer, where $U_{n}$ provides coarse, wideband analog phase shifting and $p_{n,l}$ allows for fine-grained digital control.

For simplicity in the subsequent optimization problem, we assume that this digital beamformer remains constant within the transmission block for any given direction *n*. Thus, we set $p_{n,l}=p_{n}$ for all l=1,…,L, and our goal is to optimize a single, time-invariant digital beamformer $p_{n}$ for each direction.

For the receive module, the received signal includes the signal received by the radar and the signal received by the suspect receiver. The received signal at the legitimate monitor *E* consists of suspicious signals, radar detection echoes, radar clutter, and noise. The received signal at the legitimate monitor *E* for the $\theta_{n}$ direction can be expressed as

(2) $$\begin{array}{c} y_{e}\left(l\right)=\underset{\mathrm{suspicious}\;\mathrm{signal}}{\underset{︸}{{\tilde{U}}_{n}^{H}h_{se}\sqrt{p_{s}}s\left(l\right)}}+\underset{\mathrm{radar}\;\mathrm{echo}}{\underset{︸}{{\tilde{U}}_{n}^{H}A_{n}U_{n}p_{n,l}c_{n}\left(l\right)}}\\ +\underset{\mathrm{radar}\;\mathrm{clutter}}{\underset{︸}{{\tilde{U}}_{n}^{H}\sum_{j}A_{j}U_{n}p_{n,l}c_{n}\left(l\right)}}+\underset{\mathrm{noise}}{\underset{︸}{n(l)}}, \end{array}$$

where ${\tilde{U}}_{n}=\left[{\tilde{u}}_{n,1},\ldots ,{\tilde{u}}_{n,M}\right]\in C^{N\times M}$ represents the analog receive beamforming matrix, while $U_{n}$ and $p_{n,l}$ are the previously defined analog and digital transmit beamformers at *E*, respectively. Furthermore, $p_{s}$ is the corresponding transmit power from *S*, and $A_{j}$ represents the channel matrix for the *j*-th clutter source. The term $n\left(l\right)∼\mathrm{CN}\left(0,\sigma^{2}I\right)$ denotes additive Gaussian white noise (AWGN) with $\sigma^{2}$ representing the noise power. When the number of antennas *N* is substantial, the radar detection system can achieve high angular resolution, which in turn significantly diminishes the clutter noise to a comparatively minimal level. Additionally, the clutter noise is inherently constrained by the maximum power limit due to the finite transmit power. By applying the central limit theorem, the aggregate effect of the clutter from multiple directions and the noise, denoted by ${\tilde{U}}_{n}^{H}\sum_{j}A_{j}U_{n}p_{n,l}+n$, can be simply treated as a Gaussian random variable with a variance of ${\tilde{\sigma }}^{2}$ [13,14].

The received signal at the suspicious receiver *D* includes suspicious signals, interfering signals from *E*, and noise. Thus the received signal at *D* for the $\theta_{n}$ direction can be written as

(3) $$\begin{array}{c} y_{d}\left(l\right)=\underset{\mathrm{suspicious}\;\mathrm{signal}}{\underset{︸}{h_{sd}\sqrt{p_{s}}s\left(l\right)}}+\underset{\mathrm{jamming}\;\mathrm{signal}}{\underset{︸}{h_{ed}^{H}U_{n}p_{n,l}c_{n}\left(l\right)}}+\underset{\mathrm{noise}}{\underset{︸}{n(l)}}, \end{array}$$

where $n∼\mathrm{CN}(0,\sigma^{2})$ denotes the AWGN, which follows a complex normal distribution with the noise variance being $\sigma^{2}$.

In order to distinguish between suspicious signals and radar echo signals, we have designed vectors ${\tilde{w}}_{s,n}$ and ${\tilde{w}}_{r,n}$ to reside in the null-spaces of ${\tilde{U}}_{n}^{H}A_{sr,n}U_{n}$ and ${\tilde{U}}_{n}^{H}h_{se}$, respectively. Thus, we can redefine ${\tilde{w}}_{s,n}≜Z_{s,n}w_{s,n}$ and ${\tilde{w}}_{r,n}≜Z_{r,n}w_{r,n}$, where $w_{s,n}$ and $w_{r,n}\in C^{(M-1)\times 1}$ are the equivalent digital receive beamforming vectors. Additionally, $Z_{s,n}$ and $Z_{r,n}\in C^{M\times (M-1)}$ are defined as the null spaces of ${\tilde{U}}_{n}^{H}A_{n}U_{n}$ and ${\tilde{U}}_{n}^{H}h_{se}$, respectively. Since the ranks of ${\tilde{U}}_{n}^{H}A_{sr,n}U_{n}$ and ${\tilde{U}}_{n}^{H}h_{se}$ are both 1, $Z_{s,n}$ and $Z_{r,n}$ are always present. This null-space design approach effectively mitigates the primary interference between the radar echo signals and suspicious signals.

With the interference between the radar and communication signals properly mitigated through the null-space projection design, we are now able to quantitatively evaluate the performance of both functionalities. To this end, we introduce multiple SINR expressions that correspond to different receiving roles and tasks within the system. These SINRs reflect the system’s ability to perform eavesdropping, communication disruption, and accurate radar localization simultaneously. Then we can obtain the SINRs of the system, each corresponding to different functionalities.

- ${\mathrm{SINR}}_{E}$: the SINR at the legitimate monitor *E* for decoding the suspicious communication signal from *S* to *D*. This measures *E*’s ability to eavesdrop on the signal from *S*.
- ${\mathrm{SINR}}_{D}$: the SINR at the suspicious receiver *D*, which receives the communication from *S* and interference from *E*. This reflects the quality of *D*’s reception, where interference from *E* degrades *D*’s signal, improving *E*’s eavesdropping ability.
- ${\mathrm{SINR}}_{S,\mathrm{radar}}$: the SINR at *E* when using radar to detect the echo reflected from *S*. This measures *E*’s radar performance in detecting *S*’s position.
- ${\mathrm{SINR}}_{D,\mathrm{radar}}$: the SINR at *E* when receiving radar echoes from *D*. This reflects *E*’s ability to detect and track *D*’s location, aiding in joint sensing and decision-making.

These four SINRs are written as

(4) $$\begin{array}{cc} {\mathrm{SINR}}_{E} & =\frac{\sum_{n=1}^{N}{∥w_{s,n}^{H}Z_{s,n}^{H}{\tilde{U}}_{n}^{H}h_{se}∥}^{2}p_{s}}{\sum_{n=1}^{N}{\tilde{\sigma }}^{2}w_{s,n}^{H}w_{s,n}}, \end{array}$$

(5) $$\begin{array}{cc} {\mathrm{SINR}}_{D} & =\frac{\sum_{n=1}^{N}{∥h_{sd,n}∥}^{2}p_{s}}{\sum_{n=1}^{N}\left({∥h_{ed,n}^{H}U_{n}p_{n}∥}^{2}\right)+N\sigma^{2}}, \end{array}$$

(6) $$\begin{array}{cc} {\mathrm{SINR}}_{S,\mathrm{radar}} & =\frac{\sum_{n=1}^{N}{∥w_{s,r,n}^{H}Z_{s,r,n}^{H}{\tilde{U}}_{n}^{H}A_{sr,n}U_{n}p_{n}∥}^{2}}{\sum_{n=1}^{N}{\tilde{\sigma }}^{2}w_{s,r,n}^{H}w_{s,r,n}}, \end{array}$$

(7) $$\begin{array}{cc} {\mathrm{SINR}}_{D,\mathrm{radar}} & =\frac{\sum_{n=1}^{N}{∥w_{D,r,n}^{H}Z_{D,r,n}^{H}{\tilde{U}}_{n}^{H}A_{rd,n}U_{n}p_{n}∥}^{2}}{\sum_{n=1}^{N}{\tilde{\sigma }}^{2}w_{D,r,n}^{H}w_{D,r,n}}, \end{array}$$

where $A_{sr}$ denotes the matrix of detection channels between radar and suspicious transmitters, and $A_{rd}$ denotes the matrix of detection channel between radar and suspicious receiver. Additionally, ${\mathrm{SIN}R}_{E}$ and ${\mathrm{SIN}R}_{D}$ are calculated over one scan period.

In our primary model, we assume ideal self-interference (SI) cancellation for the full-duplex monitor *E*. However, in practical implementations, imperfect analog and digital cancellation would result in residual self-interference (RSI). This RSI manifests as an additional noise-like term at the receiver of *E*. Consequently, the SINR for the eavesdropping link, ${\mathrm{SIN}R}_{E}$, would be modified. Specifically, the power of the RSI, denoted as $\sigma_{\mathrm{RSI}}^{2}$, is added to the noise and clutter term in the denominator.

The structure of our bilevel optimization problem remains intact with this modification, meaning our proposed solution methodology is still applicable. To quantitatively address the level of tolerable RSI, we can directly analyze the condition for successful eavesdropping, ${\mathrm{SINR}}_{E}>{\mathrm{SINR}}_{D}$. By incorporating the RSI power, $\sigma_{\mathrm{RSI}}^{2}$, into the denominator of ${\mathrm{SIN}R}_{E}$ as defined in (4), this condition becomes     

(8) $$\frac{\sum_{n=1}^{N}{∥w_{s,n}^{H}Z_{s,n}^{H}{\tilde{U}}_{n}^{H}h_{se}∥}^{2}p_{s}}{\left(\sum_{n=1}^{N}{\tilde{\sigma }}^{2}w_{s,n}^{H}w_{s,n}\right)+\sigma_{\mathrm{RSI}}^{2}}>{\mathrm{SINR}}_{D}$$

Rearranging this inequality allows us to establish a clear upper bound on the tolerable RSI power:

(9) $$\sigma_{\mathrm{RSI}}^{2}<\frac{\sum_{n=1}^{N}{∥w_{s,n}^{H}Z_{s,n}^{H}{\tilde{U}}_{n}^{H}h_{se}∥}^{2}p_{s}}{{\mathrm{SINR}}_{D}}-\sum_{n=1}^{N}{\tilde{\sigma }}^{2}w_{s,n}^{H}w_{s,n}$$

This inequality provides a precise, quantitative answer: the tolerable RSI power is not a fixed value but is contingent upon the strength of the eavesdropped signal at *E*, the target’s link quality (${\mathrm{SINR}}_{D}$), and the receiver’s original noise floor. Our framework is effective as long as the system’s SI cancellation capability can meet this condition.

### 2.2. Problem Formulation

The core challenge in this ISAC system is to jointly optimize two coupled objectives: maximizing the probability of successful eavesdropping and ensuring radar localization accuracy. Owing to the inherent hierarchy of these tasks—surveillance being the primary mission, with sensing as a concurrent requirement—we model this problem using a bilevel optimization framework.

**Figure 2:**
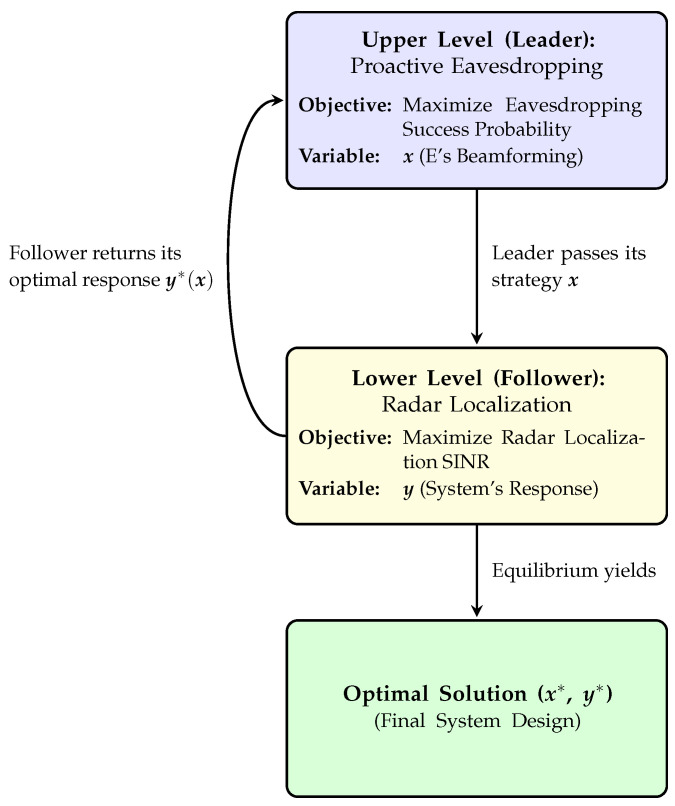
The bilevel optimization framework.

In this framework, the upper-level objective aims to maximize the probability of successful eavesdropping, while the lower-level objective is to optimize the localization performance of the radar on suspicious transmitters. This leader–follower structure reflects the practical separation of mission priority and allows each subtask to adapt to the outcome of the other. To provide a clear visual representation of this bilevel interaction, we illustrate the proposed framework in Figure 2.

To evaluate the success of the upper-level objective—legitimate eavesdropping—we adopt the SINR-based decoding criterion established in [15]. Specifically, if ${\mathrm{SINR}}_{E}<{\mathrm{SINR}}_{D}$, the legitimate monitor is incapable of error-free information eavesdropping. On the other hand, if ${\mathrm{SINR}}_{E}>{\mathrm{SINR}}_{D}$, the legitimate monitor can ensure the reliability of its information decoding. Based on this principle, we define the following indicator function to characterize the occurrence of successful eavesdropping:

(10) $$\begin{array}{c} Y=\left\{\begin{array}{cc} 1, & {\mathrm{SINR}}_{E}\geq {\mathrm{SINR}}_{D},\\ 0, & {\mathrm{SINR}}_{E}<{\mathrm{SINR}}_{D}. \end{array}\right. \end{array}$$

Therefore, the upper-level objective is to maximize the successful eavesdropping probability, denoted as $P_{\mathrm{eav}}=P\left({\mathrm{SINR}}_{E}\geq {\mathrm{SINR}}_{D}\right)$. While deriving a closed-form expression for $P_{\mathrm{eav}}$ is intractable for our complex system, it is well known that the probability of correct detection (or decoding) is a monotonically increasing function of the received SINR, as detailed in classical detection theory [16,17]. Thus, maximizing the SINR gap, $\Delta_{\mathrm{SINR}}={\mathrm{SINR}}_{E}-{\mathrm{SINR}}_{D}$, provides a direct and tractable proxy for maximizing the true probability of success. So, the upper-level objective can be written as

(11) $$\max_{U_{n},p_{n},{\tilde{U}}_{n}}\frac{\sum_{n=1}^{N}{∥w_{s,n}^{H}Z_{s,n}^{H}{\tilde{U}}_{n}^{H}h_{se}∥}^{2}\;p_{s}}{\sum_{n=1}^{N}{\tilde{\sigma }}^{2}w_{s,n}^{H}w_{s,n}}\;-\;\frac{\sum_{n=1}^{N}{∥h_{sd,n}∥}^{2}p_{s}}{\sum_{n=1}^{N}{∥h_{ed,n}^{H}U_{n}p_{n}∥}^{2}\;+\;N\sigma^{2}}.$$

Then, because the lower-level objective is the localization performance of radar at the suspicious transmitter, we can formulate it as the ${\mathrm{SINR}}_{S,\mathrm{radar}}$. Combining the upper-level and the lower-level objectives, and adding some constraints, we can rewrite the bilevel problem as

(12a) $$\begin{array}{ccc} \;\;\;\;\;\;\;\; & \;\;\;\max_{U_{n},p_{n},{\tilde{U}}_{n}} & \frac{\sum_{n=1}^{N}\;{∥w_{s,n}^{H}Z_{s,n}^{H}{\tilde{U}}_{n}^{H}h_{se}∥}^{2}\;\;p_{s}}{\sum_{n=1}^{N}{\tilde{\sigma }}^{2}w_{s,n}^{H}w_{s,n}}\;-\;\frac{\sum_{n=1}^{N}{∥h_{sd,n}∥}^{2}p_{s}}{\sum_{n=1}^{N}\;{∥h_{ed,n}^{H}U_{n}p_{n}∥}^{2}\;\;+\;N\sigma^{2}},\;\;\;\;\; \end{array}$$

(12b) $$\begin{array}{ccc} \;\;\;\;\;\;\;\; & \;\;\;s.t. & U_{n},p_{n},{\tilde{U}}_{n}\in \underset{U_{z,n},p_{z,n},{\tilde{U}}_{z,n}}{argmax}\frac{\;\sum_{n=1}^{N}\;{∥w_{s,r,n}^{H}Z_{s,r,n}^{H}{\tilde{U}}_{z,n}^{H}A_{sr,n}U_{z,n}p_{z,n}∥}^{2}}{\sum_{n=1}^{N}{\tilde{\sigma }}^{2}w_{s,r,n}^{H}w_{s,r,n}},\;\;\;\; \end{array}$$

(12c) $$\begin{array}{ccc} \;\;\;\;\;\;\;\; & \;\;\;\;\; & \;\;\;{∥U_{z,n}p_{z,n}∥}^{2}\leq p_{\max},\;\left|U_{z,n}\left(i,j\right)\right|=1, \end{array}$$

(12d) $$\begin{array}{ccc} \;\;\;\;\;\;\;\; & \;\;\;\;\; & \;\;\;\frac{{∥w_{D,r,n}^{H}Z_{D,r,n}^{H}{\tilde{U}}_{z,n}^{H}A_{rd,n}U_{z,n}p_{z,n}∥}^{2}}{\;\sum_{n=1}^{N}{\tilde{\sigma }}^{2}w_{D,r,n}^{H}w_{D,r,n}}\geq \gamma_{r},\;\forall n, \end{array}$$

where the variables $U_{z,n},p_{z,n},{\tilde{U}}_{z,n}$ represent the same physical quantities as $U_{n},p_{n},{\tilde{U}}_{n}$, respectively. These variables are introduced merely for convenience in the subsequent algorithm exposition, and their inclusion helps facilitate the optimization process. The constraints ${∥U_{z,n}p_{z,n}∥}^{2}\leq p_{\max}$ reflect the power limits, while the condition ${\mathrm{SINR}}_{D,\mathrm{radar}}\geq \gamma_{r}$ guarantees the effectiveness of radar detection.

The above problem is a bilevel problem. Here, the lower-level problem aims to maximize the ${\mathrm{SIN}R}_{S,\mathrm{radar}}$ (12b) under the power limitation and other constraints. Within the set of the optimal solution to the lower-level problem, the upper-level problem aims to maximize the ${\mathrm{SINR}}_{E}-{\mathrm{SINR}}_{D}$ (12a). Unlike the traditional single-level problem, we need to obtain the set of optimal solutions at the lower level and then bring in the upper level to find the optimal solution at the upper level. However, since bilevel optimization requires a convex lower-level problem, which is not satisfied in our case, we cannot apply it directly. In the following, we transform the original problem into an equivalent form that can be tackled more effectively.

## 3. Proposed Algorithm

In this section, we first use the Rayleigh quotient to put the problem in its simplest form. Then, we decouple our problems and introduce penalty terms so that we can develop a solution for the lower-level problem with convex underlying goals. Finally, we introduce the bilevel algorithm to solve the problem.

### 3.1. Simplification and Reformulation of Problem (12)

We now consider the optimization problem in lower-level problem (12b). Let us define

(13) $$\begin{array}{cc} w_{s} & ≜{\left[w_{s,r,1}^{H},w_{s,r,2}^{H},\ldots ,w_{s,r,N}^{H}\right]}^{H}\in C^{(M-1)N\times 1}, \end{array}$$

(14) $$\begin{array}{cc} h_{s} & ≜\left[\begin{array}{c} {\left(Z_{s,r,1}^{H}A_{sr,1}^{H}U_{z,1}p_{z,1}\right)}^{H}\\ {\left(Z_{s,r,2}^{H}A_{sr,2}^{H}U_{z,2}p_{z,2}\right)}^{H}\\ \vdots \\ {\left(Z_{s,r,N}^{H}A_{sr,N}^{H}U_{z,N}p_{z,N}\right)}^{H} \end{array}\right]\in C^{(M-1)N\times 1}. \end{array}$$

Then, we can transform the problem (12b) into the following problem:

(15) $$\begin{array}{c} \max_{w_{s}}\frac{{∥w_{s}^{H}h_{s}∥}^{2}}{{\tilde{\sigma }}^{2}w_{s}^{H}w_{s}}. \end{array}$$

To solve this, we first analyze the numerator. Since $w_{s}^{H}h_{s}$ is a scalar, its squared norm can be expanded as:

(16) $$\begin{array}{cc} {∥w_{s}^{H}h_{s}∥}^{2} & =\left(w_{s}^{H}h_{s}\right){\left(w_{s}^{H}h_{s}\right)}^{H}=\left(w_{s}^{H}h_{s}\right)\left(h_{s}^{H}w_{s}\right)\\ & =w_{s}^{H}\left(h_{s}h_{s}^{H}\right)w_{s}. \end{array}$$

By substituting this back into (15), the optimization problem becomes

(17) $$\max_{w_{s}}\;\frac{w_{s}^{H}\left(h_{s}h_{s}^{H}\right)w_{s}}{w_{s}^{H}w_{s}}.$$

This expression is a generalized Rayleigh quotient of the form $\frac{x^{H}Ax}{x^{H}x}$. Here, the matrix $A=h_{s}h_{s}^{H}$ is rank-1 and, importantly, Hermitian, since $A^{H}={\left(h_{s}h_{s}^{H}\right)}^{H}=h_{s}h_{s}^{H}=A$. The maximum value of this quotient is the largest eigenvalue of *A*, and the optimal vector $w_{s}$ is the corresponding principal eigenvector. For a rank-1 matrix like $h_{s}h_{s}^{H}$, the principal eigenvector is simply $h_{s}$ itself. This observation justifies the formulation and leads directly to Lemma 1.

**Lemma 1.**  *Based on the Rayleigh quotient* [18]*, a closed-form expression for the optimal $w_{s}$ can be achieved as*

(18) $$\begin{array}{c} w_{s}=\frac{h_{s}}{{\tilde{\sigma }}^{2}}. \end{array}$$

Since problem (15) is a typical Rayleigh quotient maximization problem, we insert Equation (18) into problem (15) and then substitute the original forms of vector $h_{s}$ and vector $w_{s}$ into the newly obtained equation. Then we can formulate the new problem as follows:

(19) $$\begin{array}{c} \max_{U_{z,n},p_{z,n},{\tilde{U}}_{z,n}}\frac{\sum_{n=1}^{N}{∥Z_{s,r,n}^{H}{\tilde{U}}_{z,n}^{H}A_{sr,n}U_{z,n}p_{z,n}∥}^{2}}{{\tilde{\sigma }}^{2}}. \end{array}$$

The objective function in (19) is non-convex and involves highly coupled variables, making it difficult to solve directly. To make the problem tractable, we employ a penalty-based decoupling strategy, a standard technique for handling complex constraints in optimization theory [19]. The core idea is to transform a problem with hard equality constraints into an approximately equivalent problem by adding quadratic penalty terms to the objective function, which penalize any deviation from the original constraints.

Following this approach, we introduce several auxiliary variables to decouple the complex expressions. Let us define

(20) $$\begin{array}{cc} X & ≜Z_{s,r,n}^{H}{\tilde{U}}_{n}^{H}A_{sr,n}U_{n}p_{n},Y≜U_{n}p_{n}, \end{array}$$

(21) $$\begin{array}{cc} X_{z} & ≜Z_{s,r,n}^{H}{\tilde{U}}_{z,n}^{H}A_{sr,n}U_{z,n}p_{z,n},Y_{z}≜U_{z,n}p_{z,n}. \end{array}$$

Here, the variables without the subscript ‘*z*’ are for the upper level, while those with ‘*z*’ are their counterparts in the lower-level problem, introduced for convenience in the algorithm. By incorporating these definitions and their corresponding penalty terms, we can reformulate the lower-level problem as:

(22) $$\begin{array}{ccc} \;\;\;\;\;\;\;\; & \;\;\;\;\; & \;\;\;U_{n},p_{n},{\tilde{U}}_{n}\in \underset{U_{z,n},p_{z,n},{\tilde{U}}_{z,n},X_{z},Y_{z}}{argmax}\;\;\;\;\frac{\sum_{n=1}^{N}{∥X_{z}∥}^{2}}{{\tilde{\sigma }}^{2}}-\mu {∥Y_{z}-U_{z,n}p_{z,n}∥}^{2}\\ \;\;\;\;\;\;\;\; & \;\;\; & \;\;\;-\mu {∥X_{z}-Z_{s,r,n}^{H}{\tilde{U}}_{z,n}^{H}A_{sr,n}U_{z,n}p_{z,n}∥}^{2},\;\;\;\;\\ \;\;\;\;\;\;\;\; & \;\;\; & \;\;\;s.t.\;\;\;{∥U_{z,n}p_{z,n}∥}^{2}\leq p_{\max},\left|U_{z,n}\left(i,j\right)\right|=1,\\ \;\;\;\;\;\;\;\; & \;\;\;\;\; & \;\;\;\frac{\sum_{n=1}^{N}{∥Z_{D,r,n}^{H}{\tilde{U}}_{z,n}^{H}A_{rd,n}Y_{z}∥}^{2}}{{\tilde{\sigma }}^{2}}\geq \gamma_{r},\\ \;\;\;\;\;\;\;\; & \;\;\;\;\; & \;\;\;X_{z}=Z_{s,r,n}^{H}{\tilde{U}}_{z,n}^{H}A_{sr,n}U_{z,n}p_{z,n},Y_{z}=U_{z,n}p_{z,n}. \end{array}$$

Similarly, we can employ (18) in the upper-level problem (12a) and combine the obtained result with Equation (22) where maximizing the objective in the lower-level problem is converted into minimizing the corresponding negative objective. Then we can obtain the formulated bilevel problem as follows:   

(23a) $$\begin{array}{ccc} \;\;\;\;\;\;\;\; & \;\;\;\min_{U_{n},p_{n},{\tilde{U}}_{n}} & \frac{\sum_{n=1}^{N}{∥h_{sd,n}∥}^{2}p_{s}}{\sum_{n=1}^{N}\left({∥h_{ed,n}^{H}Y∥}^{2}\right)+N\sigma^{2}}-\frac{\sum_{n=1}^{N}{∥Z_{s,n}^{H}{\tilde{U}}_{n}^{H}h_{se,n}∥}^{2}p_{s}}{{\tilde{\sigma }}^{2}},\;\;\;\;\; \end{array}$$

(23b) $$\begin{array}{ccc} \;\;\;\;\;\;\;\; & \;\;\;s.t. & U_{n},p_{n},{\tilde{U}}_{n}\in \;\;\;\;\;\underset{U_{z,n},p_{z,n},{\tilde{U}}_{z,n},X_{z},Y_{z}}{argmax}\;\;\;\;-(\frac{\sum_{n=1}^{N}{∥X_{z}∥}^{2}}{{\tilde{\sigma }}^{2}}-\mu {∥Y_{z}-U_{z,n}p_{z,n}∥}^{2}\\ \;\;\;\;\;\;\;\; & \;\;\; & -\mu {∥X_{z}-Z_{s,r,n}^{H}{\tilde{U}}_{z,n}^{H}A_{sr,n}U_{z,n}p_{z,n}∥}^{2}),\;\;\;\; \end{array}$$

(23c) $$\begin{array}{ccc} \;\;\;\;\;\;\;\; & \;\;\;\;\; & \;\;\;{∥U_{z,n}p_{z,n}∥}^{2}\leq p_{\max},\;\left|U_{z,n}\left(i,j\right)\right|=1, \end{array}$$

(23d) $$\begin{array}{ccc} \;\;\;\;\;\;\;\; & \;\;\;\;\; & \;\;\;\frac{\sum_{n=1}^{N}{∥Z_{D,r,n}^{H}{\tilde{U}}_{z,n}^{H}A_{rd,n}Y_{z}∥}^{2}}{{\tilde{\sigma }}^{2}}\geq \gamma_{r},\;\forall n, \end{array}$$

(23e) $$\begin{array}{ccc} \;\;\;\;\;\;\;\; & \;\;\;\;\; & \;\;\;X=Z_{s,r,n}^{H}{\tilde{U}}_{n}^{H}A_{sr,n}U_{n}p_{n},Y=U_{n}p_{n}, \end{array}$$

(23f) $$\begin{array}{ccc} \;\;\;\;\;\;\;\; & \;\;\;\;\; & \;\;\;X_{z}=Z_{s,r,n}^{H}{\tilde{U}}_{z,n}^{H}A_{sr,n}U_{z,n}p_{z,n},Y_{z}=U_{z,n}p_{z,n}. \end{array}$$

### 3.2. Proposed Algorithm to the Bilevel Problem

To solve the constrained bilevel optimization problem in our ISAC system, we adopt a first-order penalty-based approach inspired by recent advances in bilevel optimization [20]. Although the lower-level subproblem in our formulation is convex, the upper-level objective involves non-convex constraints, making the bilevel structure difficult to handle directly using standard techniques such as KKT-based reformulations or implicit differentiation.

To address this, we reformulate the bilevel problem by explicitly defining the upper-level objective f(x,y), the lower-level objective $\tilde{f}\left(z,y\right)$, and the lower-level constraints $\tilde{g}\left(x,z\right)$. These functions encode the physical objectives of our system. f(x,y) reflects the performance metric related to the legitimate monitor, such as signal reception or eavesdropping capability; $\tilde{f}\left(z,y\right)$ quantifies the radar sensing quality; $\tilde{g}\left(x,z\right)$ captures the power and SINR constraints associated with the radar operations. With these definitions, the original bilevel problem can be expressed in the standard form:

(24) $$\begin{array}{c} f=\min f\left(x,y\right)\\ s.t.\;y\in \underset{z}{\arg\;\min}\left\{\tilde{f}\left(x,z\right)|\tilde{g}\left(x,z\right)\leq 0\right\}. \end{array}$$

The above structure matches the setting studied in [20], where the lower-level problem is a convex problem but the upper-level problem may be a non-convex one. Under such conditions, a penalty reformulation is proposed to approximate the bilevel problem using a minmax formulation, which can be solved with traditional methods.

In our specific case, since all decision variables appear in either *y* or *z*, and no variable is independently assigned to *x*, we set x←∅, $y\leftarrow \{U_{n},p_{n},{\tilde{U}}_{n},X,Y\}$ and $z\leftarrow \{U_{z,n},p_{z,n},{\tilde{U}}_{z,n},X_{z},Y_{z}\}$. The bilevel problem is thus written as

(25) $$\begin{array}{c} f=\min f\left(y\right)\\ s.t.\;y\in \underset{z}{\arg\min}\left\{\tilde{f}\left(z\right)|\tilde{g}\left(z\right)\leq 0\right\}. \end{array}$$

To proceed with solving the bilevel problem formulated in Equation (25), we follow the first-order penalty approach introduced in [20]. Specifically, we introduce a penalty function to handle the lower-level constraint by incorporating it into the objective.

(26) $${\tilde{P}}_{\mu }\left(x,z\right)=\tilde{f}\left(x,z\right)+\mu {∥{\left[\tilde{g}\left(x,z\right)\right]}_{+}∥}^{2},$$

where μ>0 is a penalty parameter and ${\left[\cdot \right]}_{+}$ denotes the component-wise maximum with zero. This penalized formulation approximates the lower-level constrained problem by smoothing the feasible region.

Based on this, the bilevel problem can be approximately reformulated as

(27) $$f_{\mu }=\min_{x,y}\left\{f\left(x,y\right)|y\in \arg\min_{z}{\tilde{P}}_{\mu }\left(x,z\right)\right\}.$$

To further eliminate the constraint $y\in \underset{z}{\mathrm{argmin}}{\tilde{P}}_{\mu }\left(z\right)$, we consider the value function of the penalized lower-level problem. Let $V_{\mu }:=\min_{z}{\tilde{P}}_{\mu }\left(x,z\right)$ denote the minimum value of the penalized lower-level objective. Then, the constraint $y\in \arg\min_{z}{\tilde{P}}_{\mu }\left(x,z\right)$ is equivalent to enforcing $\min_{z}{\tilde{P}}_{\mu }\left(x,z\right)=V_{\mu }$. This motivates a value-function-based penalization of the form

(28) $$f\left(y\right)+\rho \left({\tilde{P}}_{\mu }\left(x,y\right)-V_{\mu }\right),$$

where ρ>0 is an outer-level penalty parameter. As shown in [20], minimizing this relaxed objective yields a solution that asymptotically satisfies the original bilevel constraints as ρ→∞. From $\underset{z}{min}{\tilde{P}}_{\mu }\left(x,z\right)=V_{\mu }$, we have

(29) $$\min_{x,y}f\left(x,y\right)+\rho \left({\tilde{P}}_{\mu }\left(x,y\right)-\min_{z}{\tilde{P}}_{\mu }\left(x,z\right)\right),$$

which can be further written as the following minmax problem:

(30) $$\min_{x,y}\max_{z}P_{\rho ,\mu }\left(x,y,z\right),$$

where the penalized objective is defined by

(31) $$P_{\rho ,\mu }\left(x,y,z\right):=f\left(x,y\right)+\rho \left({\tilde{P}}_{\mu }\left(x,y\right)-{\tilde{P}}_{\mu }\left(x,z\right)\right).$$

We now turn to the structure of the functions involved in the objective and constraints. It is worth noting that the constant modulus constraint, $\left|U_{z,n}\left(i,j\right)\right|=1$, is handled differently from other constraints. Instead of introducing a corresponding penalty term, we enforce this constraint directly in our algorithm by parameterizing the elements of the analog beamformer as complex exponentials. This ensures the constraint is always satisfied by construction. The remaining functions and constraints are handled by the penalty framework. Specifically, the upper-level objective f(y) and the lower-level function $\tilde{f}\left(z\right)$ are split into components as follows:

(32) $$\begin{array}{c} f\left(y\right)=f_{1}\left(y\right)=\frac{\sum_{n=1}^{N}{∥h_{sd,n}∥}^{2}p_{s}}{\sum_{n=1}^{N}{∥h_{ed,n}^{H}Y∥}^{2}+N\sigma^{2}}-\frac{\sum_{n=1}^{N}{∥Z_{s,n}^{H}{\tilde{U}}_{n}^{H}h_{se,n}∥}^{2}p_{s}}{{\tilde{\sigma }}^{2}},\\ \tilde{f}\left(z\right)={\tilde{f}}_{2}\left(z\right)=-\frac{\sum_{n=1}^{N}{∥X_{z}∥}^{2}}{{\tilde{\sigma }}^{2}}+\mu {∥Y_{z}-U_{z,n}p_{z,n}∥}^{2}+\mu {∥X_{z}-Z_{s,r,n}^{H}{\tilde{U}}_{z,n}^{H}A_{sr,n}U_{z,n}p_{z,n}∥}^{2}. \end{array}$$

The function $f_{1}\left(y\right)$ represents the upper-level performance metric associated with the legitimate monitor reception quality or eavesdropping capability, while $f_{2}\left(y\right)$ quantifies the penalty term that comes from the radar sensing quality, which we want to minimize to reduce interference.

Similarly, the constraints are divided as

(33) $$g_{1}\left(z\right)={∥U_{z,n}p_{z,n}∥}^{2}-p_{\max},\;g_{2}\left(z\right)=\gamma_{r}-\frac{\sum_{n=1}^{N}{∥Z_{D,r,n}^{H}{\tilde{U}}_{z,n}^{H}A_{rd,n}Y_{z}∥}^{2}}{{\tilde{\sigma }}^{2}}.$$

The constraints $g_{1}\left(y\right)$ and $g_{2}\left(y\right)$ capture the power and SINR constraints for the radar operations. Specifically, $g_{1}\left(y\right)$ ensures that the power consumption does not exceed the maximum power $p_{\max}$, and $g_{2}\left(y\right)$ captures the SINR constraints to guarantee radar sensing quality.

The decomposition of the objective and constraints into separate components for the upper and lower levels is a direct consequence of the penalty-based reformulation. This allows us to handle the minimization and maximization of the functions separately, which simplifies the optimization process and ensures that the penalization of constraint violations is smoothly incorporated. This provides a natural way to handle the original bilevel problem as a minmax problem. Therefore, the function in (31) can be decomposed using the additive structure of *f* and $\tilde{f}$, yielding

(34) $$\begin{array}{cc} P_{\rho ,\mu }\left(x,y,z\right) & =f_{1}\left(x,y\right)+f_{2}\left(x\right)+\rho \left({\tilde{f}}_{1}\left(x,y\right)-{\tilde{f}}_{1}\left(x,z\right)\right)\\ \; & \;+\rho \mu \left(∥{\left[\tilde{g}\left(x,y\right)\right]}_{+}{∥}^{2}-{∥{\left[\tilde{g}\left(x,z\right)\right]}_{+}∥}^{2}\right)\\ \; & \;+\rho \left({\tilde{f}}_{2}\left(y\right)-{\tilde{f}}_{2}\left(z\right)\right). \end{array}$$

Given that $P_{\rho ,\mu }\left(x,y,z\right)$ is non-convex in (x,y), concave in *z*, and built from smooth and proximally well-behaved functions, the corresponding minmax problem can be equivalently reformulated as

(35) $$\min_{x}\max_{y}\left\{h(x,y)+p(x)-q(y)\right\},$$

where the individual terms are defined as

(36) $$\begin{array}{cc} p(x) & =\rho {\tilde{f}}_{2}\left(x\right),\;q\left(y\right)=\rho {\tilde{f}}_{2}\left(y\right),\\ h(x,y) & =f_{1}\left(x\right)+\rho \mu \left(∥{\left[{\tilde{g}}_{1}\left(x\right)\right]}_{+}{∥}^{2}+{∥{\left[{\tilde{g}}_{2}\left(x\right)\right]}_{+}∥}^{2}\right)\\ \; & \;-\rho \mu \left(∥{\left[{\tilde{g}}_{1}\left(y\right)\right]}_{+}{∥}^{2}+{∥{\left[{\tilde{g}}_{2}\left(y\right)\right]}_{+}∥}^{2}\right). \end{array}$$

Thus, the original bilevel optimization problem has been successfully reformulated as a structured minmax problem. This reformulation enables the application of the traditional methods for solving bilevel problems. To efficiently solve the resulting minmax problem, we design an iterative algorithm that alternately updates the variables *x* and *y* while dynamically adjusting the penalty parameters ρ and μ. It is important to note that this algorithmic framework is robust; as established in our system model, practical imperfections such as RSI do not alter the fundamental problem structure, thus ensuring the applicability of the proposed method. The detailed procedure is outlined as Algorithm 1.

To elaborate, Algorithm 1 is designed to solve the structured minmax problem by alternately updating the variables *y* and *x*. At each iteration, the algorithm first updates *y* by solving the inner maximization problem. Specifically, it minimizes the negative of the objective function, that is −(h(x,y)+p(x)−q(y)), to approximate the maximum with respect to *y* given the current estimate of *x*. Once the optimal *y* corresponding to the current *x* is obtained, the algorithm substitutes it into the objective and proceeds to update *x* by solving the outer minimization problem. In this way, the algorithm progressively minimizes the outer objective while accounting for the adversarial behavior of *y*. Meanwhile, the penalty parameters ρ and μ are dynamically adjusted during the iterations to gradually enforce constraint satisfaction and improve solution stability. This alternating optimization strategy enables the algorithm to approach a saddle point of the original minmax formulation and effectively solve the reformulated bilevel problem.

**Convergence and Feasibility Analysis.** Regarding the convergence and feasibility of Algorithm 1, it is important to note that for a non-convex problem such as this, guaranteeing convergence to a global optimum is generally intractable. Our objective is instead to find a high-quality stationary point. The feasibility of the solution is ensured by the penalty method itself; as the penalty parameters ρ and μ are progressively increased during the iterations, the solution is driven towards the feasible set of the original constrained problem [20]. While a formal convergence proof for our specific alternating algorithm is beyond the scope of this paper, this structure is a standard and effective heuristic designed to find a stationary point of the reformulated minmax problem. The practical effectiveness of this convergence is validated in our simulation results.

**Computational Complexity Analysis.** The overall computational complexity of the proposed Algorithm 1 is dominated by the iterative updates of variables *x* and *y*. In each outer iteration, the algorithm solves two main subproblems: a maximization with respect to *y* and a minimization with respect to *x*. Assuming a gradient-based method is used to solve each subproblem for a fixed number of inner steps, the cost per iteration is primarily determined by the gradient computation of the functions h(x,y), p(x), and q(y). The dimensions of the variables *x* and *y* are mainly determined by the number of antennas *N* and RF chains *M*. Consequently, the complexity of computing the gradient for each step involves matrix–vector multiplications and scales polynomially with these parameters. Let $T_{\mathrm{outer}}$ be the number of outer iterations and $T_{\mathrm{inner}}$ be the number of inner gradient steps for each subproblem. The total computational complexity can be characterized as $O(T_{\mathrm{outer}}\cdot T_{\mathrm{inner}}\cdot \mathrm{poly}\left(N,M\right))$, where poly(N,M) represents the cost of a single gradient evaluation. As our simulation results demonstrate, the algorithm is practically efficient, converging within a few hundred outer iterations, which validates the effectiveness of this approach.

## 4. Simulation Results

In this section, we present the simulation results that demonstrate the superiority of our bilevel algorithm.
**Algorithm 1** The proposed method for the minmax problem (35)**Input:**N,M (N≫M), $x^{0},y^{0}$, ρ>0, μ=0.5, tolerance $=10^{-20}$, maxIters =3000, t=0, etc. **for** i=0,1,2,… **do**     **for** j=0,1,2,… **do**         t←0         **while** $∥x^{t+1}-x^{t}∥+∥y^{t+1}-y^{t}∥\geq$ tolerance **and** t≤ maxIters **do**            Update $y^{t+1}$ by solving:                $\min_{y}\left\{-(h\left(x^{t},y\right)+p\left(x^{t}\right)-q\left(y\right))\right\}$            Update $x^{t+1}$ by solving:                $\min_{x}\left\{h\left(x,y^{t+1}\right)+p\left(x\right)-q\left(y^{t+1}\right)\right\}$            Update ρ, μ            t←t+1         **end while**     **end for** **end for**

### 4.1. Simulation Setup

In our simulations, we adopt a hybrid analog–digital architecture where the numbers of antennas and RF chains are set to N=16 and M=4, respectively. The inter-antenna spacing is set to half the wavelength (d=0.5λ). The main beam is directed toward an angle of $\theta_{n}=\pi /4$, and other angles $\theta_{1}$ and $\theta_{2}$ are randomly generated around π/6 and π/4, respectively, to simulate the beam directions.

The noise power is set as $\sigma =\tilde{\sigma }=3$W. The impact of noise on performance is not considered separately. Additionally, the maximum transmit power is fixed at $P_{s}=10$ dBm. The gain factor $\beta_{n}$ is set to 1.2. The constraint of the radar’s reception of signals from the suspicious receiver *D*, denoted as $\gamma_{r}$, is specified in relevant sections.

The locations of the suspicious transmitter *S*, suspicious receiver *D*, and the legitimate monitor *S* are set as follows: *S* is placed at the origin (0,0)m, *D* is located at (0,60)m, and *S* is positioned at (80,0)m.

The wireless channels between S-E, S-D, and E-D are generated based on a composite fading model that includes both large-scale and small-scale effects. The large-scale fading is modeled as a distance-dependent path loss using the logarithmic model.

(37) $$L\left(d\right)=30+36\log_{10}\left(d\right)\;\left(\mathrm{dB}\right),$$

where *d* is the Euclidean distance in meters between the transmitter and receiver. The corresponding path loss gain is given by $G\left(d\right)=10^{-L(d)/10}$. The small-scale fading follows a Rician distribution, where the Rician K-factors $K_{\mathrm{SE}}$, $K_{\mathrm{SD}}$, and $K_{\mathrm{ED}}$ are set to $10^{0.3}$, $10^{0.8}$, and $10^{0.6}$, corresponding to 3dB, 8dB, and 6dB, respectively. The Rician K-factors represent the power ratio between the line-of-sight (LoS) and non-line-of-sight (NLoS) components. A higher η indicates a stronger LoS dominance. The final channel matrices are computed as the product of the large-scale fading gain and the Rician fading component.

To supplement the SINR metric and provide a more direct assessment of radar performance, we also evaluate the probability of detection ($P_{D}$). Following established methodologies in detection theory [21,22], we calculate the $P_{D}$ by mapping our simulated ${\mathrm{SIN}R}_{S,\mathrm{radar}}$ results for a given constant Probability of False Alarm ($P_{FA}$). This allows for a direct translation from the SINR, which our algorithm optimizes, to a more practical operational metric. For this calculation, we set the false alarm rate to $P_{FA}=10^{-3}$ and the number of pulses to L=4.

### 4.2. Performance Verification

To evaluate the performance of the proposed bilevel algorithm, we compare it with a benchmark scheme as described below.

**Conventional Constrained Single-Level Scheme.** To highlight the advantages of our bilevel approach, we compare it against a more conventional single-level constrained optimization scheme that aims to maximize the upper-level objective, namely, the active eavesdropping capability, while incorporating the lower-level goal, the radar localization performance, as a constraint. Specifically, this scheme solves the following single-level problem:

(38a) $$\begin{array}{cc} \max_{U_{n},p_{n},{\tilde{U}}_{n}}\;\; & \frac{\sum_{n=1}^{N}{∥Z_{s,n}^{H}{\tilde{U}}_{n}^{H}h_{se,n}∥}^{2}p_{s}}{{\tilde{\sigma }}^{2}}-\frac{\sum_{n=1}^{N}{∥h_{sd,n}∥}^{2}p_{s}}{\sum_{n=1}^{N}{∥h_{ed,n}^{H}Y∥}^{2}+N\sigma^{2}} \end{array}$$

(38b) $$\begin{array}{cc} s.t.\;\; & {\mathrm{SINR}}_{S,\mathrm{radar}}\geq \gamma_{r1}, \end{array}$$

(38c) $$\begin{array}{cc} \; & {\mathrm{SINR}}_{D,\mathrm{radar}}\geq \gamma_{r2}, \end{array}$$

(38d) $$\begin{array}{cc} \; & ∥U_{z,n}p_{z,n}{∥}^{2}\leq p_{\max},\;\left|U_{z,n}\left(i,j\right)\right|=1,\;\forall n,i,j.\; \end{array}$$

Because the radar performance is enforced via the constraint in (38b), the resulting beamforming solution is functionally radar-aware. This provides a fair and meaningful comparison against our bilevel formulation, allowing us to evaluate which optimization philosophy—hierarchical co-optimization versus constrained single-level optimization—achieves a better trade-off between the two objectives.

**Figure 3:**
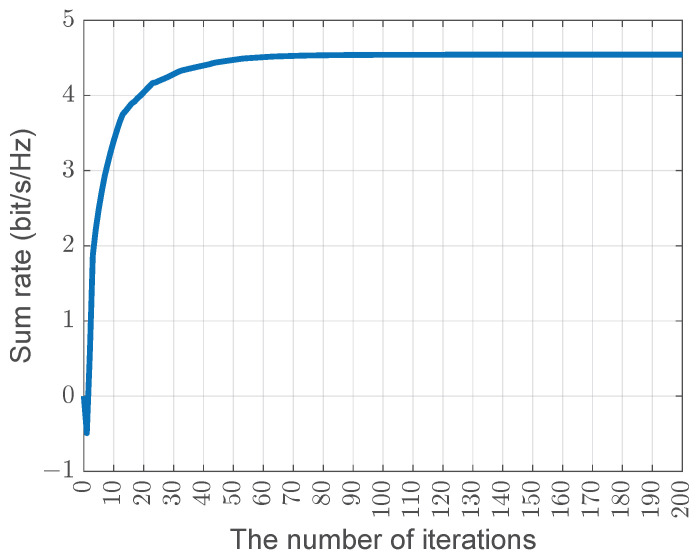
Convergence behavior of the bilevel algorithm.

Figure 3 illustrates the convergence behavior of the bilevel algorithm, showing how the radar’s sum rate changes with the number of iterations. From the figure, it is evident that the algorithm converges quickly, reaching near convergence after approximately 80 to 100 iterations. After this point, the sum rate stabilizes, indicating that the algorithm achieves efficient convergence with relatively few iterations.

**Figure 4:**
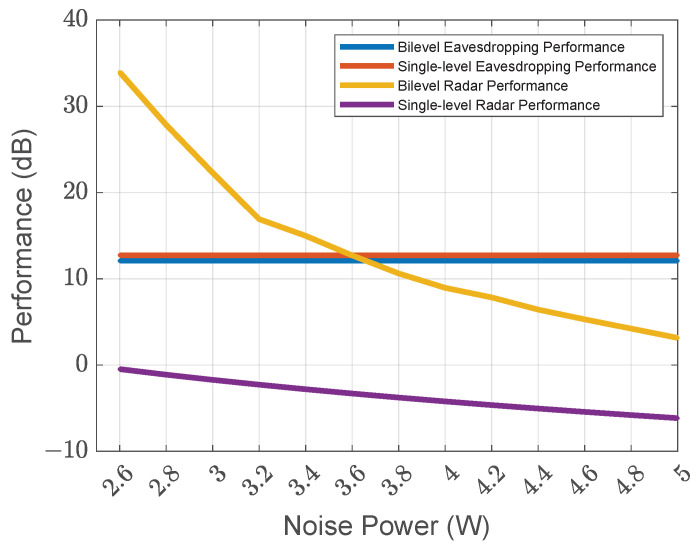
Performance versus noise power.

We also compare the performance of the bilevel algorithm against single-level algorithm in terms of the noise power parameter as shown in Figure 4. Although the single-level algorithm (represented by the red curve) slightly outperforms the bilevel algorithm in eavesdropping performance (represented by the blue curve), the bilevel algorithm demonstrates superior overall performance by optimizing both the upper-level and lower-level objectives. As we can see, the radar performance of the bilevel algorithm (represented by the yellow curve) surpasses the radar performance of the single-level algorithm (represented by the purple curve). As the noise power increases, the bilevel algorithm maintains a higher radar performance, showing its robustness in handling noise.

**Figure 5:**
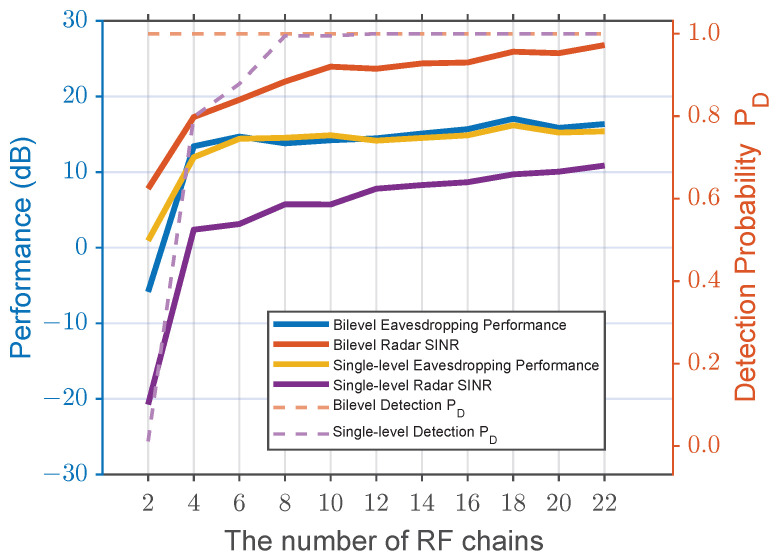
Performance versus the number of RF chains.

Next, the performance with respect to the number of RF chains is illustrated in Figure 5. As the number of RF chains increases, both eavesdropping and radar performance improve for both the bilevel and single-level algorithms. However, the key insight here is the radar performance (represented by the red curve for the bilevel algorithm and the purple curve for the single-level algorithm) clearly demonstrates that the bilevel algorithm maintains a significantly higher advantage. Meanwhile, the dashed curves in Figure 5 depict the corresponding detection probabilities. Consistent with the SINR trends, the bilevel scheme sustains an almost unit-level $P_{D}$ across the entire RF-chain range, whereas the single-level scheme starts below 0.5 and only approaches near-perfect detection once M≥8, further underscoring the bilevel algorithm’s robustness in preserving radar reliability.

Additionally, as the number of RF chains increases, the eavesdropping performance of the bilevel algorithm (represented by the blue curve) gradually surpasses that of the single-level algorithm (represented by the yellow curve). This shows that the bilevel optimization approach better balances radar and eavesdropping objectives, resulting in superior radar performance especially as the number of RF chains increases. This growing advantage can be attributed to the increased spatial degrees of freedom for precoding; with more RF chains, the bilevel algorithm can better leverage spatial multiplexing to craft more sophisticated beamformers that effectively nullify interference while simultaneously satisfying the radar performance constraints.

**Figure 6:**
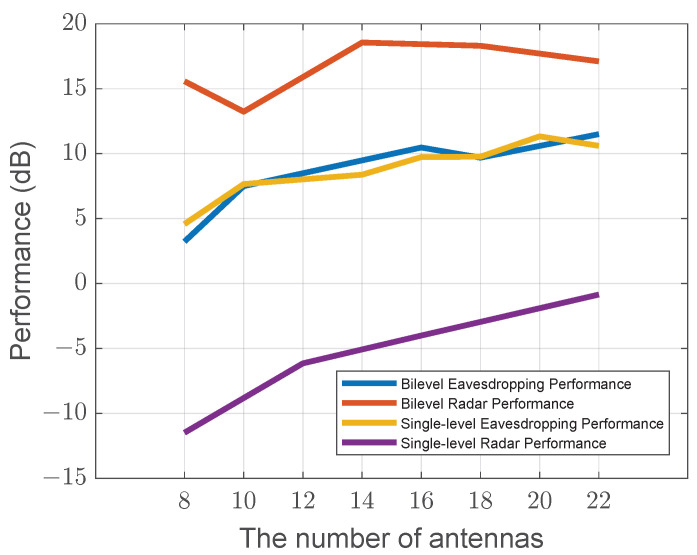
Performance versus the number of antennas.

Additionally, in Figure 6, we analyze the performance with respect to the number of antennas. As the number of antennas increases, it is evident that the eavesdropping performance (represented by the blue curve for the bilevel algorithm and the yellow curve for the single-level algorithm) shows minimal difference between the bilevel and single-level algorithms. Both algorithms exhibit a general improvement as the number of antennas grows, with the bilevel algorithm maintaining slightly better performance in some cases. However, the key distinction between the two approaches lies in radar performance. The bilevel algorithm significantly outperforms the single-level algorithm in radar detection, demonstrating a superior ability to leverage additional antennas for better radar functionality.

Although we observe that, with a higher number of antennas, the bilevel radar performance starts to slightly decrease, this counter-intuitive trend is not an algorithmic flaw but a feature of sophisticated trade-off management. This phenomenon can be attributed to two factors: first, a power dilution effect where the fixed total power is spread across more antennas, and second, a strategic decision by the bilevel optimizer. With more spatial degrees of freedom available, the algorithm intelligently determines that a marginal sacrifice in the secondary radar objective can be traded for a more significant gain in the primary eavesdropping objective. This reduction is still less pronounced compared to the substantial improvements seen in the single-level radar performance. The bilevel optimization method still maintains its edge by achieving a more balanced and optimal use of antennas across both radar and eavesdropping tasks, ensuring that the trade-off does not heavily compromise the overall performance.

**Figure 7:**
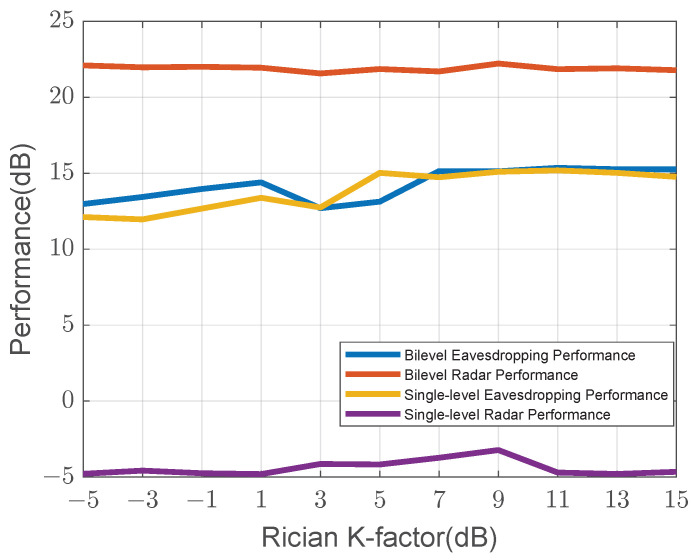
Performance versus Rician K-factor.

Finally, we evaluate the robustness of our proposed algorithm under varying channel conditions by sweeping the Rician K-factor from −5 dB to 15 dB, with the results shown in Figure 7. The K-factor indicates the ratio of the LoS power to the NLoS power; a lower K-factor indicates a more challenging, scattering-rich environment, while a higher K-factor represents a more stable, LoS-dominant channel. As observed, the key advantage of the proposed bilevel algorithm lies in its robust radar performance. The radar SINR (represented by the red curve) remains consistently high across the entire range of the K-factor, demonstrating that our algorithm successfully protects the lower-level objective regardless of the channel quality, which varies from NLoS-dominant (low K) to LoS-dominant (high K). Regarding the eavesdropping performance, both schemes exhibit similar behavior, with performance generally improving as the channel becomes more predictable with a higher K-factor.

Across various system parameters (noise power, RF chains, antenna count) and channel conditions (Rician K-factor), our scheme consistently demonstrates a superior ability to manage the trade-off between radar localization and proactive eavesdropping.

### 4.3. Discussion on Model Assumptions and Limitations

In this section, we elaborate on the key assumptions of our system model to clarify their suitability and limitations, thereby contextualizing our results.

**Gaussian Clutter and Noise Model:** The assumption that aggregate clutter and noise follow a Gaussian distribution is a foundational and practical simplification in the radar signal processing literature. This is justified by the central limit theorem, particularly when clutter arises from a large number of independent, randomly distributed scatterers. We acknowledge, however, that this model has limitations. In environments characterized by a few dominant, "spiky" clutter sources (e.g., large buildings), the interference may exhibit heavier tails and could be more accurately described by other statistical models, such as the K-distribution or Weibull distribution.

**Rician Fading Channel Model:** Our use of the Rician fading model is standard for wireless channels that include a dominant LoS path alongside scattered multipath components. This is highly relevant for ISAC scenarios, where a stable LoS path is often crucial for reliable sensing. The Rician K-factor allows us to parameterize the strength of this LoS component. To explicitly validate our algorithm’s performance across different channel realities, our robustness analysis with respect to the K-factor as shown in Figure 7 demonstrates that our algorithm maintains strong radar performance even in challenging low-K (NLoS-dominant) conditions.

**Perfect Channel State Information (CSI):** Following the prevailing practice in ISAC research [23], we assume perfect CSI at the transmitter. This idealized setting establishes an unequivocal performance upper bound and allows the intrinsic advantage of our bilevel optimization structure to emerge without the confounding influence of channel-estimation errors. Nevertheless, CSI in real deployments is inevitably imperfect. According to [23], uncertainty in CSI induces a fundamental trade-off between communication throughput and the accuracy of channel-state estimation, thereby motivating robust physical-layer designs. Several mature techniques can be embedded into our framework to mitigate such uncertainty, including worst-case robust optimization—which bounds the estimation error within an ellipsoidal set and, via the S-procedure, yields a tractable semidefinite program [24], chance-constrained formulations that ensure probabilistic performance guarantees when only error statistics are available, and distributionally robust methods that hedge against model mismatch. Integrating these robust counterparts into the following problem while preserving the upper-level resource-allocation strategy constitutes a primary direction of our future work.

## 5. Conclusions

In this paper, a bilevel optimization algorithm has been developed for ISAC systems, aiming to maximize the active eavesdropping probability while ensuring high radar localization performance. We reformulated the problem by using the Rayleigh quotient, decoupling the objectives and introducing penalty terms. This allowed us to apply a bilevel algorithm to optimize both beamforming matrices. Extensive simulation results demonstrate that our algorithm effectively maintains high radar localization accuracy while achieving nearly the same active eavesdropping probability as existing methods, showcasing its superior performance in ISAC scenarios. Future work will consider extending the proposed framework to multi-user scenarios and enhancing robustness under imperfect channel conditions.

## Data Availability

The original contributions presented in this study are included in the article. Further inquiries can be directed to the corresponding authors.
